# Supplementary material for: Improved Diabetic Wound Healing by EGF Encapsulation in Gelatin-Alginate Coacervates
Source: Pharmaceutics. 2020 Apr 8;12(4):334. doi: 10.3390/pharmaceutics12040334 (PMC7238057; doi:10.3390/pharmaceutics12040334)
Supplement: Supplementary file 1 [file pharmaceutics-12-00334-s001.pdf]

pharmaceutics

Supplementary Materials: Improved Diabetic Wound Healing by EGF Encapsulation in Gelatin-Alginate Coacervates

Seonghee Jeong, ByungWook Kim, Minwoo Park, Eunmi Ban, Soo-Hyeon Lee and Aeri Kim

Table S1. Body weights of mice used for cytokine analysis.

Group	Animal No.	0 day (10/25)	3 day (10/28)
Negative	1	23.61	-
	2	23.24	22.71
	3	23.92	24.12
	4	23.24	23.98
	Mean	23.50	23.60
	SD	0.33	0.78
EGF-PM	1	25.28	25.86
	2	22.48	22.60
	3	24.38	23.73
	4	24.20	24.20
	Mean	24.09	24.10
	SD	1.17	1.35
EGF-Coa	1	21.12	20.69
	2	21.94	21.45
	3	22.63	22.49
	4	22.98	22.66
	Mean	22.17	21.82
	SD	0.82	0.93

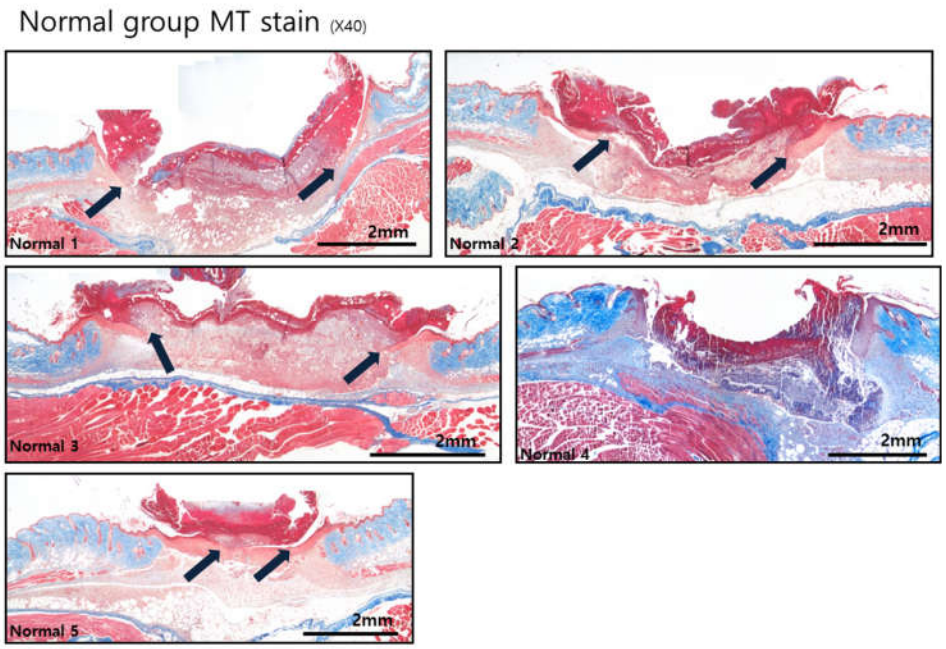

(a)

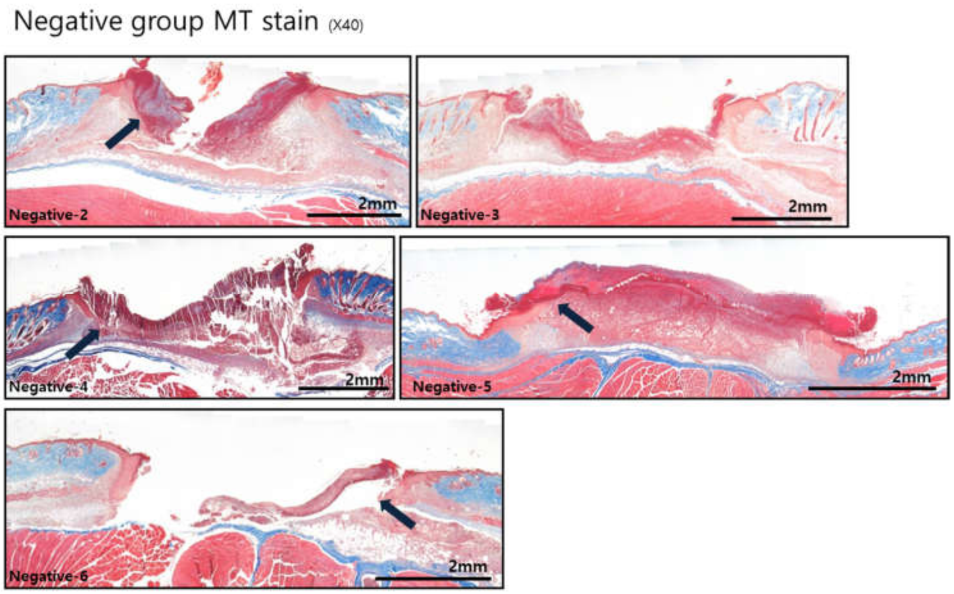
Negative group MT stain (X40)
Negative-2
2mm
Negative-3
2mm
Negative-4
2mm
Negative-5
2mm
Negative-6
2mm

(b)

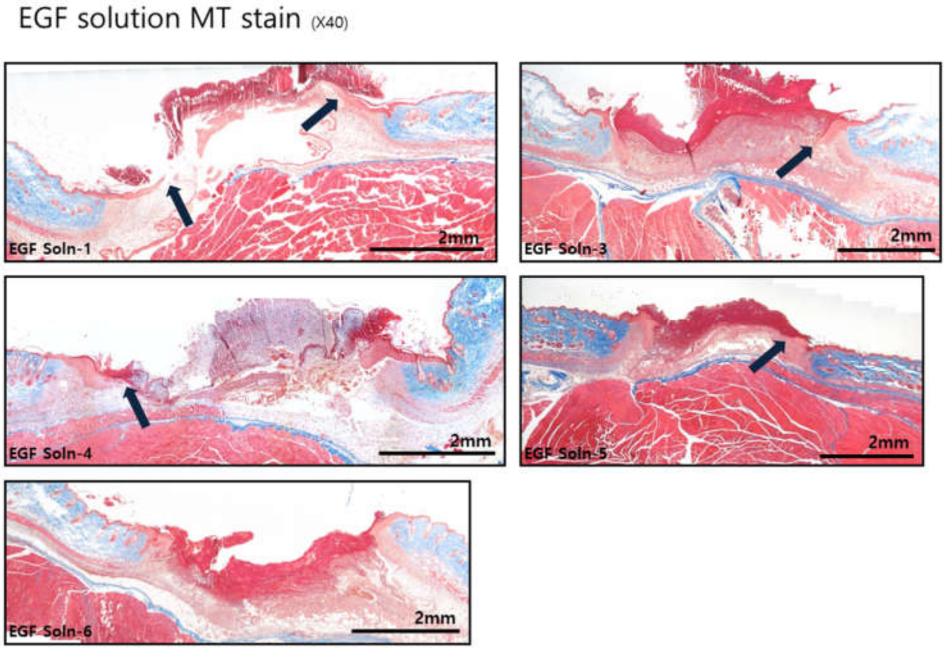
EGF solution MT stain (X40)
EGF Soln-1
2mm
EGF Soln-3
2mm
EGF Soln-4
2mm
EGF Soln-5
2mm
EGF Soln-6
2mm

(c)

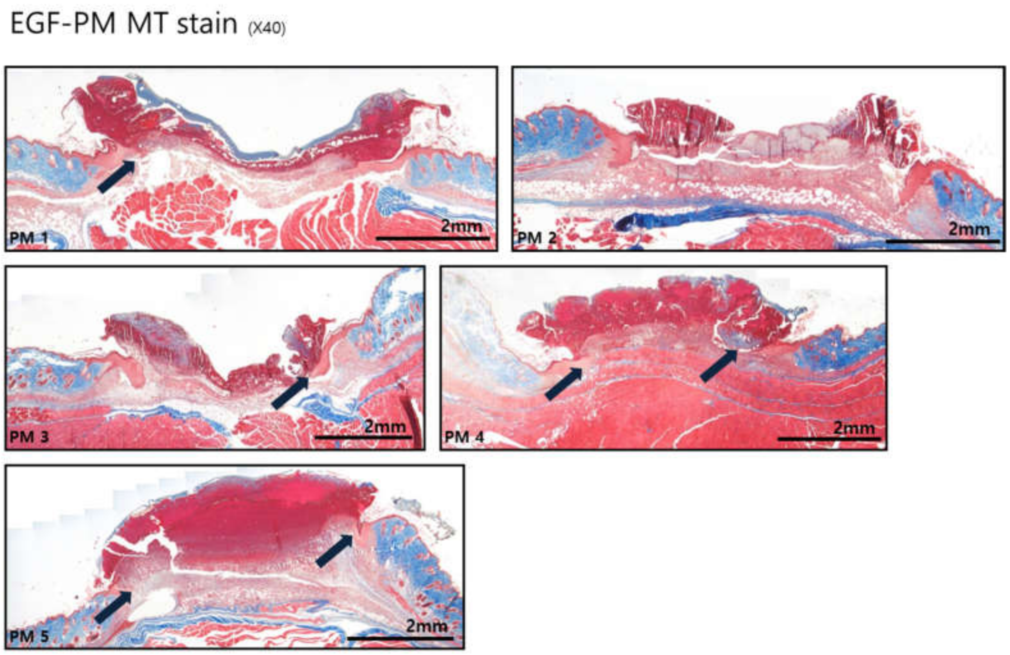

(d)

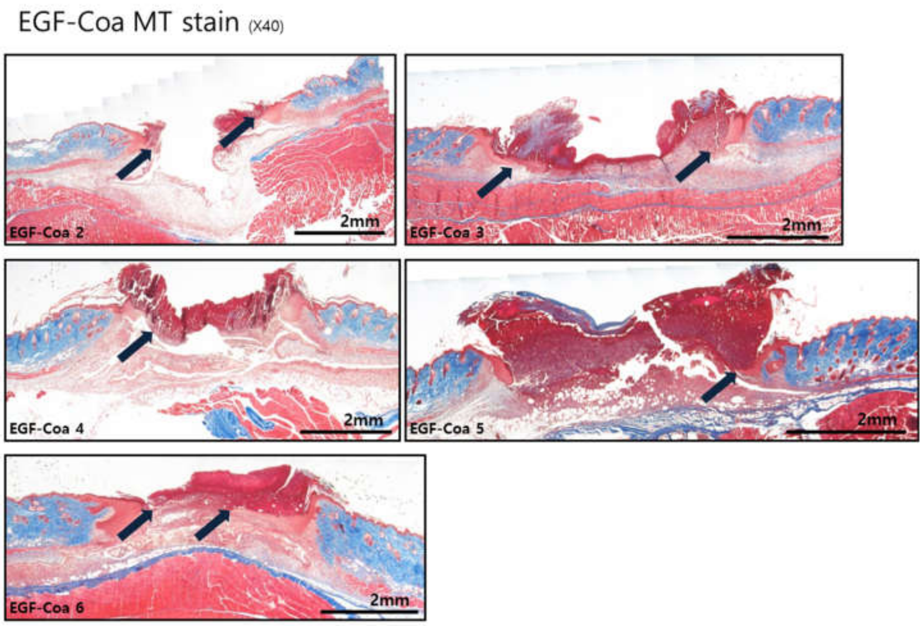

(e)

Figure S1. Histological images of wound beds with Masson's trichrome staining. (**a**) Normal (non-diabetic mice), (**b**) Negative (diabetic mice) treated with PBS, (**c**), (**d**), and (**e**) Diabetic mice treated with EGF solution, EGF-PM and EGF-Coa. Black arrows indicate the pointed fronts of horizontal migration of keratinocytes along the surface of newly formed granulation tissue.

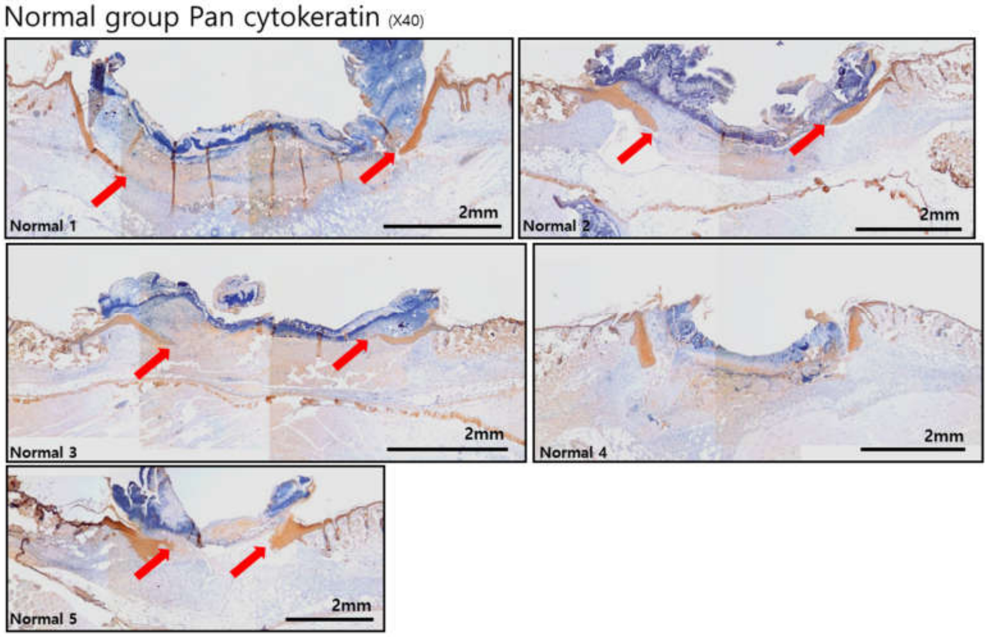
Normal group Pan cytokeratin (X40)
Normal 1
2mm
Normal 2
2mm
Normal 3
2mm
Normal 4
2mm
Normal 5
2mm

(a)

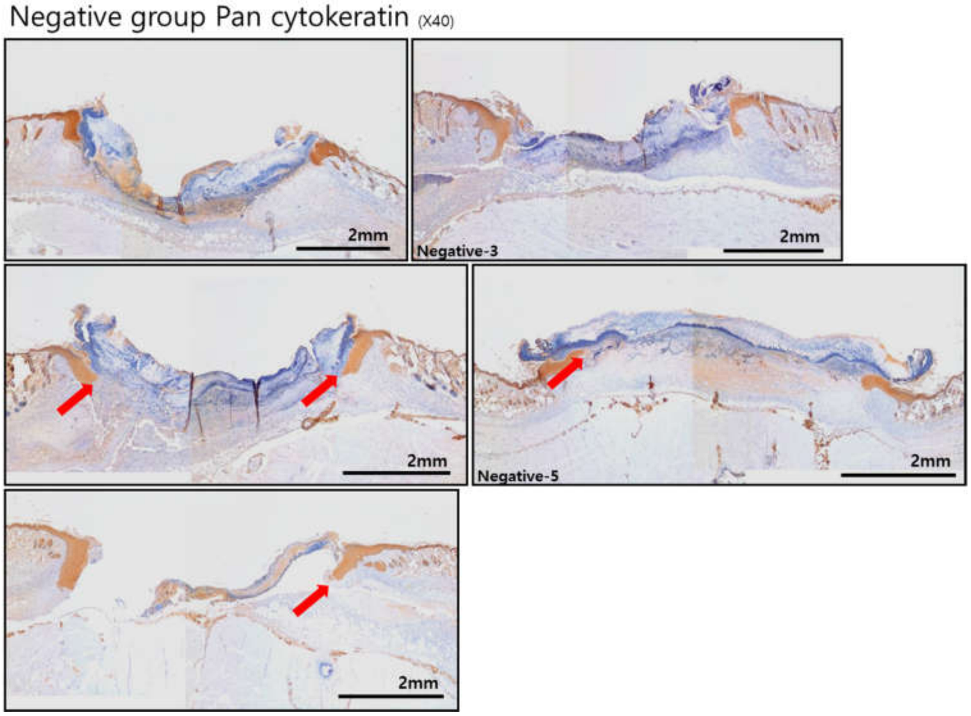
Negative group Pan cytokeratin (X40)
2mm
Negative-3
2mm
2mm
Negative-5
2mm
2mm

(b)

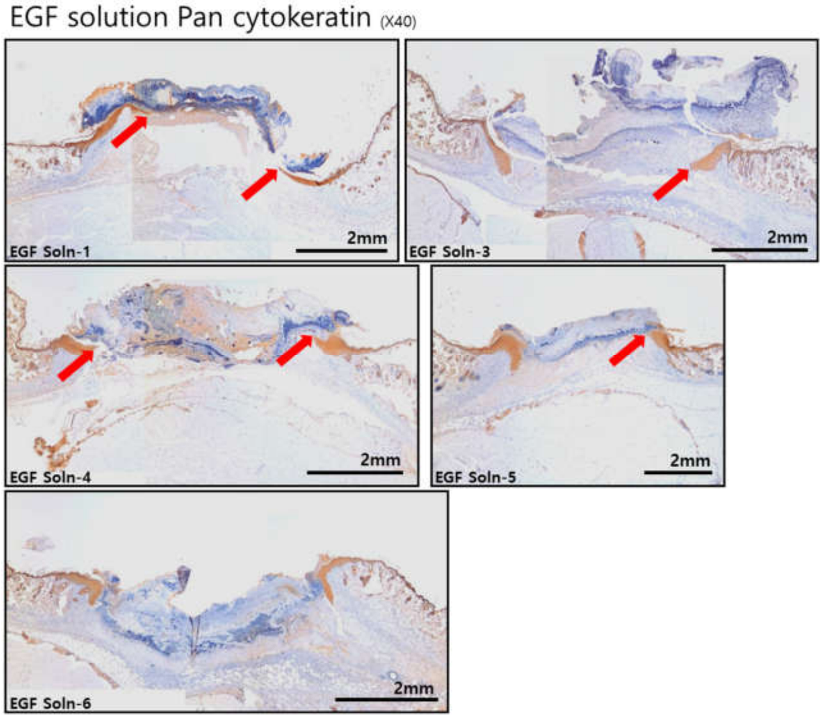
EGF solution Pan cytokeratin (X40)
EGF Soln-1
2mm
EGF Soln-3
2mm
EGF Soln-4
2mm
EGF Soln-5
2mm
EGF Soln-6
2mm

(c)

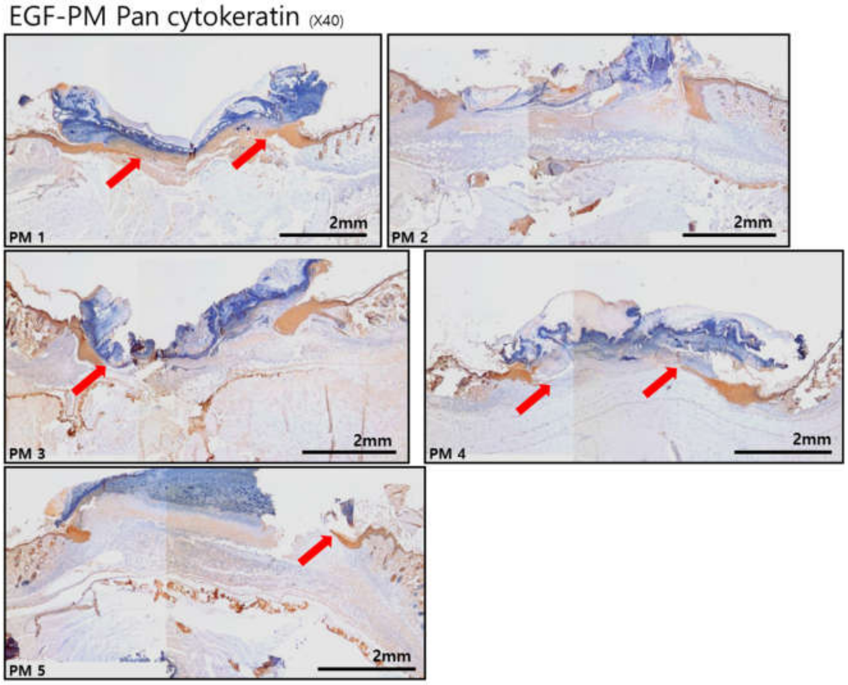
EGF-PM Pan cytokeratin (X40)
PM 1
2mm
PM 2
2mm
PM 3
2mm
PM 4
2mm
PM 5
2mm

(d)

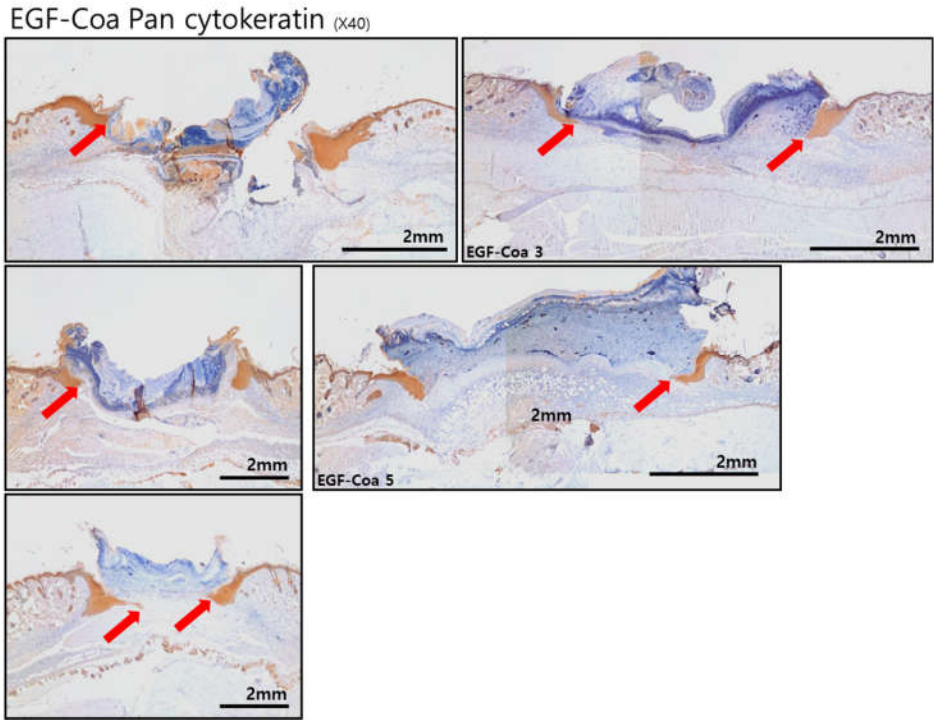

(e)

Figure S2. Histological images of wound beds with pancytokeratine staining: (**a**) Normal (non-diabetic mice), (**b**) Negative (diabetic mice) treated with PBS, (**c**), (**d**), and (**e**) Diabetic mice treated with EGF solution, EGF-PM and EGF-Coa. Red arrows indicate the pointed fronts of horizontal migration of keratinocytes along the surface of newly formed granulation tissue.
